# Menopause Hot Flashes and Molecular Mechanisms Modulated by Food-Derived Nutrients

**DOI:** 10.3390/nu16050655

**Published:** 2024-02-26

**Authors:** Ewa Forma, Karina Urbańska, Magdalena Bryś

**Affiliations:** 1 Department of Cytobiochemistry, Faculty of Biology and Environmental Protection, University of Lodz, Pomorska 141/143, 90-236 Lodz, Poland; magdalena.brys@biol.uni.lodz.pl; 2 Faculty of Medicine, Medical University of Lodz, 90-419 Lodz, Poland; karina.urbanska@stud.umed.lodz.pl

**Keywords:** hot flashes, menopause, food-derived nutrients, molecular cell signaling pathways

## Abstract

The causes of vasomotor symptoms, including hot flashes, are not fully understood, may be related to molecular factors, and have a polygenic architecture. Nutrients and bioactive molecules supplied to the body with food are metabolized using various enzymatic pathways. They can induce molecular cell signaling pathways and, consequently, activate effector proteins that modulate processes related to hot flashes in menopausal women. In this review, we analyzed the literature data from the last 5 years, especially regarding genome-wide association study (GWAS) analysis, and selected molecular factors and cell signaling pathways that may potentially be related to hot flashes in women. These are the kisspeptin-GnRH pathway, adipocyte-derived hormones, aryl hydrocarbon receptor signaling, catechol estrogens and estrogen sulfotransferase, inflammatory and oxidative stress biomarkers, and glucose availability. Then, single compounds or groups of food ingredients were selected that, according to experimental data, influence the course of the discussed molecular pathways and thus can be considered as potential natural therapeutic agents to effectively reduce the troublesome symptoms of menopause in women.

## 1. Introduction

Menopause is a normal biological process that occurs in women at an average age of 50 and is defined as the 12-month period of amenorrhea in the absence of any other physiological or pathological causes [1,2]. It is preceded by the menopausal transition (termed the climacteric or perimenopause), which occurs between the onset of irregular menstruation cycles and menopause [3]. Clinical manifestations of menopausal transition include menstrual disorder, hot flashes, sweating, insomnia, palpitation, vertigo, headache, tinnitus, problems with memory, inability to focus, mood changes (anxiety and depression), changes in body weight and composition, skin changes and genitourinary atrophy symptoms. Approximately two-thirds of women suffer from these menopause-related symptoms [2,3,4,5].

One of the well-known classic symptoms of the menopausal transition is hot flashes. Hot flashes are vasomotor symptoms, which occur in more than 75% of menopausal women [2,6,7,8]. The medium duration of hot flashes is approximately four years, with some lasting as long as 20–30 years [2,9,10]. Hot flashes are described as transitory episodes of heat sensations, flushing and excessive sweating in the face and chest. The sensation lasts from 2 to 4 min and is associated with palpitation, anxiety, irritability, and night sweats. The frequency of hot flash episodes varies from occasional attacks in a week or day to at least one every hour [2,8,10,11]. Hot flashes are often associated with impairments in quality of life, loss of productivity, depressed mood, embarrassment, fatigue, anxiety, sleep disturbance, and possibly even poorer memory function and social isolation [4,7,12]. The physiological and molecular mechanism of hot flashes is still incompletely known. Hot flashes during menopause are based on complex endocrine, neuroendocrine and epigenetic mechanisms [13]. The hot flashes are triggered by a small elevation in core body temperature, which causes activation of the sympathetic nervous system by peripheral vasodilation and increased activity of sweat glands. This mechanism may be associated with the response of the hypothalamus to decreased estrogen levels and modulation of the neurotransmitter activity (serotonin and noradrenaline) [4].

Hormone therapy is the most effective treatment for hot flashes, but it is not always possible to use [14]. Many women are looking for alternative medicines for relieving their menopausal symptoms [4]. Since the molecular mechanisms responsible for hot flashes have not yet been fully understood, we are trying to identify less common signaling pathways with high probability involved in this process, based on the latest literature. Our indications are largely hypothetical because we rely on, for example, the results of GWAS analyses, which always precede experimental data. Substances contained in food can certainly influence the mechanisms of hot flashes and modulate their symptoms. In the presented review, based on the latest literature, we not only identify further potential molecular mechanisms responsible for the occurrence of hot flashes but also analyze natural food ingredients that may modulate these molecular mechanisms and signaling pathways.

## 2. Molecular Factors Associated with Hot Flashes and Nutrients

The etiology of vasomotor symptoms, including hot flashes, is unclear, but it could include genetics and have a polygenic architecture. Food-derived nutrients and bioactive molecules, on the other hand, are metabolized through different molecular pathways, and these pathways vary from person to person. This metabolic diversity is, among other things, the result of genetic and epigenetic variability [14,15].

Sex steroid hormone levels are associated with hot flashes, but their relation with these symptoms is moderate, and other mechanisms are important. Thermoregulatory mechanisms mediated by the sympathetic and parasympathetic systems appear to play a role in hot flashes. As a result of their activity, the thermoneutral zone is narrowed and, consequently, heat is dissipated. The thermoneutral zone in the body is responsible for the functioning of thermoregulatory homeostatic mechanisms [10,16,17]. The summary of the results of recent in vitro and in vivo studies on nutritional compounds modulating cell signaling pathways that may potentially be involved in mechanisms causing hot flashes is presented in Table 1.

## 3. The Kisspeptin-GnRH Pathway

The results of the systematic review by Crandall et al. [15] extensively analyze the genetic background of hot flashes. A genome-wide association study (GWAS) analysis, which is discussed in this article, revealed 14 SNPs associated with hot flashes with a significant probability. All SNPs were located in the tachykinin receptor 3 gene (TARC3), acting as a neurokinin B (NKB) receptor. In postmenopausal women, hypertrophy of neurons expressing NKB and estrogen receptor α (ERα) mRNA was observed. In comparative studies in pre- and postmenopausal women, it was found that after menopause in hypertrophied neurons, the expression of neuropeptides at the mRNA level is higher. These neurons also form a denser network of axosomatic and axodendritic connections with gonadotropin-releasing hormone (GnRH) neurons. It was also found that in monkeys and rats undergoing ovariectomy, there was an increase in NKB gene expression. This process was reversed after estradiol therapy [14,31,32,33]. GnRH secretion is controlled by the functional neural network of the kisspeptin-neurokinin B-dynorphin (KNDy) arcuate nucleus system. This system is part of the estrogenic feedback mechanism in the estrogen receptor-hypothalamus scheme [34]. During menopause, the hypothalamus controls GnRH and thermoregulation through neuropeptides such as kisspeptin and neurokinin B, which are overexpressed in people with vasomotor symptoms. Administration of neurokinin B 3 receptor agonists causes hot flashes, tachycardia and increased skin temperature [11]. A detailed description of the physiology of GnRH and the involvement of the hypothalamic kisspeptin-neurokinin-dynorphin neuronal network in the regulation of GnRH secretion is included in the review article by Marques et al. [35].

As shown by Xiong et al. [19], genistein affects GnRH secretion in GT1-7 (mouse hypothalamic GnRH neuronal cell line) cells via modulating kisspeptin receptors, SIRT1 (silent information regulator 1), PKCγ (protein kinase c γ) and MKRN3 (makorin ring finger protein 3) [19]. The same researchers also showed that racemic (±)-equol increases the ability to secrete GnRH, and interfere with the expression of neurokinin B receptor, MKRN3, and SIRT1 in murine neurons [18]. S-equol is a metabolite of daidzein (dietary soy isoflavone), which is created as a result of intestinal bacterial flora action [34,35,36]. Equol has higher biological activity than daidzein, due to the fact that this compound can interact with estrogen receptors and thus induce signal transmission in the cell. The end result of these processes is the activation of the expression of specific genes and, in the translation process, the synthesis of biologically active proteins that, for example, regulate both hormone levels and oxidative stress. On average, only 45% of the population have the ability to produce equol after consuming soy. Lactic acid bacteria are a heterogeneous group that have been proposed as probiotics and microorganisms that can be used for the production of nutraceuticals. In improving technological processes in the production of food and dietary supplements, the permanent introduction of Bifidobacterium, Lactococcus, or Lactobacillus is also considered to convert daidzein into equol [37].

It is suggested that GnRH neurons may sense and integrate signals from circulating fatty acids. Tran et al. (2020) reported that the PUFA (polyunsaturated fatty acid), DHA (docosahexaenoic acid), and the SFA (saturated fatty acid palmitate) palmitate directly upregulate GnRH mRNA expression in Adult Mouse Hypothalamic GnRH/GFP (green fluorescent protein) cell line neurons [20]. In turn, Wang et al. (2020) showed that palmitate dysregulates hypothalamic function by spexin (SPX), and its receptors galanin receptor 2 (GALR2) and galanin receptor 3 (GALR3) regulation, in GnRH neurons via mechanisms involving protein kinase C (PKC), mitogen-activated protein kinases (MAPKs), and Toll-like receptor 4 (TLR4) [27].

On the other hand, undernutrition suppresses gonadotropin-releasing hormone (GnRH)/luteinizing hormone (LH) secretion. KNDy neurons coexpress neuropeptides that are stimulatory (kisspeptin and neurokinin B [NKB]) and inhibitory (dynorphin) in pulsatile GnRH/LH release, and undernutrition would reduce kisspeptin, while the expression of NKB will simultaneously inhibit the expression of dynorphin [21].

## 4. Adipocyte-Derived Hormones

In the hypothalamus, signals are exchanged between the nervous and endocrine systems, and this system of signals is created by hormones circulating in the body. Under the influence of stimuli from the hypothalamus, the pituitary gland produces hormones that regulate the functioning of other endocrine glands. In this way, changes in their secretion may influence abnormal eating behavior. The most important hormones responsible for nutritional processes are leptin, resistin, ghrelin, and insulin. Leptin is produced mainly by adipose tissue cells, and it is secreted from adipose tissue under the influence of insulin, in the period after food consumption, when the elevated glucose concentration is lowered by insulin to the physiological level. The most important target site of leptin’s action is the hunger and satiety center in the hypothalamus. There, the receptors react with leptin, as a result of which neurons stop producing the neurotransmitter, neuropeptide Y, which is an appetite stimulator. In this way, the desire to eat is inhibited. Leptin accelerates metabolism, inhibits the deposition of fat tissue, and activates its breakdown. Because the amount of leptin produced depends on the amount of fat tissue in the body, chronically elevated leptin levels in the blood are observed in overweight or obese people. In this case, the cells of the hypothalamus “get used to” the constantly elevated level of this hormone and eventually stop responding to it. This means that despite the body’s energy needs being met, the feeling of hunger and appetite is not suppressed after eating a meal [38]. As shown by Karim et al., higher ghrelin concentrations are positively correlated with a greater likelihood of hot flashes in women in the early and late postmenopausal period [39]. Based on their research, Sau et al. suggest a relationship between hot flashes and the occurrence of metabolic syndrome in women aged 40–65 [12]. Also, Kazama et al. showed that the fat mass index was positively associated with severe hot flashes, whereas the lean mass index was negatively correlated [40].

Energy metabolism dysfunction is triggered by abnormal interactions between the hypothalamus and adipose tissue. Extracellular vesicles (EVs) from adipocytes play an important role in mediating the interaction between adipocytes and hypothalamic neurons. Adipocyte-derived EVs can regulate pro-opiomelanocortin (POMC) neuron expression through the hypothalamic mammalian target of rapamycin (mTOR) signaling, thus influencing the body’s energy intake. The research was conducted on an in vitro and in vivo animal model [41].

It should be emphasized that 17β-estradiol as an anorexogenic hormone activates the genomic and nongenomic cell signaling pathway through metabolic neurons—proopiomelanocortin (POMC) neurons decreased food intake, and neuropeptide Y (NPY)/agouti-related peptide (AgRP) neurons increased food intake. 17β-estradiol, apart from feedback control of the reproductive axis, modulates autonomic functions regulated by the hypothalamus, such as energy homeostasis and body temperature. Signaling pathways involving 17β-estradiol as a ligand and ERα as a receptor are considered to be a critical element of the hypothalamic regulation of energy balance. Studies on rodents have shown that low estrogen levels correlate positively with decreased activity and increased body weight. An increased incidence of type 2 diabetes, hyperinsulinemia and obesity was observed in men with a mutation in the ERα gene, which resulted in loss of function of the protein product [42]. Although the direct relationship between the described process and vasomotor changes in women has not been studied, we indicate this mechanism and its possible involvement in generating hot flashes.

In turn, research conducted by Huang et al. (2017) indicates the relationship between hot flashes and the development of metabolic disorders. Scientists have shown that hot flashes are associated with insulin resistance in postmenopausal women and that the relationship between hot flashes and insulin resistance depends on leptin–adiponectin interaction [43].

## 5. Aryl Hydrocarbon Receptor Signaling

Moreover, candidate gene studies found significant associations between SNPs in several genes and hot flashes. These genes belong to the aryl hydrocarbon receptor pathways or are genes regulated by this transcription factor. Finally, the following genes were listed—aryl hydrocarbon receptor (AHR), aryl hydrocarbon receptor repressor (AHRR), aryl hydrocarbon receptor nuclear translocator (ARNT), catechol-O-methyltransferase (COMT), cytochrome P450 (CYP) family 1 subfamily A member 1 (CYP1 A1), CYP family 1 subfamily A member 2 (CYP1A2), CYP450 family 1 subfamily B member 1 (CYP1B1), CYP3 subfamily A member 4 (CYP3A4), and CYP19 subfamily A member 1 (CYP19A1) [13,38]. The relationships between the protein products of these genes are very complex and poorly understood at the molecular level. We will investigate the relationship between food ingredients and the activity of protein products of these genes based on literature data.

AHR, as a ligand-activated transcription factor, modulates cell transmission signals in a ligand-type-dependent, cell- and tissue-specific manner. Estrogen receptor α (ERα) and ERβ act as transcription factors and can be activated through genomic and nongenomic mechanisms by hormones, proteins with kinase activity, or other transcription factors. Cellular signaling pathways involving the AHR and ER may influence each other. Phosphorylated AHR inhibits ER activity, while ERα plays a positive role in AHR signaling. Activated AHR has also been shown to inhibit the expression of 17β-estradiol (E2)-induced genes [39,40,41,43,44,45,46,47,48]. It has been found that exogenous AHR ligands that will activate cell signal transmission may be derived from the diet and include tryptophan, indole, microbiota-derived short-chain fatty acids, triglycerides, polyamines, glucose, fructose, cholesterol, flavonoids, curcumin and carotenoids [20,21,23,24,26,49,50]. Other AHR ligands are discussed in detail in the articles of Bungsu et al. [45] and De Juan and Segura [51], with particular emphasis on indoles contained in cruciferous vegetables, but these considerations apply to cellular signal transmission in general and not to hot flashes [41,52]. Reports by Kim et al. [53] on vitamin B12 and folic acid as AHR antagonists should also be mentioned.

Variations in sex steroid metabolism genes such as CYP1B1 may occur. The function of CYP1B1 is to convert 17β-estradiol into 2-hydroextradiol-17β, which changes the balance of estradiol as the main female sex hormone. Fluctuations in estradiol levels occurring during menopause are associated with the etiology of hot flashes [15]. It has been shown that the AHR-ARNT complex (AHR nuclear translocator) binds to the xenobiotic response element (XRE) in regulatory genes, inducing specific gene expression of enzymes such as CYP1A1, CYP1A2 and CYP1B1 [54]. The association between some nutrients and CYP1B1 action is described by Shah et al. (2019) [54]. The article considers such compounds as apigenin, luteolin, quercetin, scutellarein, kaempferol, taxifolin, indole-3-carbinol, folic acid, piceatannol and compounds contained in flaxseed, green tea extracts and olive oil. Furthermore, in silico studies have shown a potential relationship between cytochrome P450 enzymes (CYP1A1 and CYP1B1) and the flavonoids isorhamnetin and pedalitin present in food [55].

## 6. Catechol Estrogens and Estrogen Sulfotransferase

The group of candidate genes selected in bioinformatics analysis also includes genes whose protein products are responsible for the metabolism of steroid hormones—estrogen receptor 1 (ESR1), hydroxysteroid 17-β dehydrogenase 1 (HSD17B1), solute carrier family 6 member 4 (SLC6A4), sulfotransferase family 1A member 1 (SULT1A1), and sulfotransferase family 1E genes [15].

Estrogen conjugates are formed by biotransformation from both endogenous and exogenous estrogens. Conjugated estrogens do not have the ability to activate ER receptors and therefore do not initiate cell signal transmission. The most active catechol estrogen (CE) conjugative pathway is methylation. CE methylation is catalyzed by catechol-O-methyltransferase (COMT), which catalyzes the transfer of methyl groups from S-adenosyl methionine to the hydroxyl groups of several catechol substrates, including the catechol estrogens [56].

Some phenolic compounds acting as substrates for catechol-O- methyltransferase (COMT)-catalyzed O-methylation can also operate as COMT inhibitors, impeding the O-methylation of a variety of catechol substrates. These compounds are present, among others, in coffee, tea and extra virgin olive oil [53,54].

Estrogen sulfotransferase (SULT1E1) is a phase II detoxifying and conjugating enzyme that catalyzes the binding of a sulfate group from 3′-phosphoadenosine-5′-phosphosulfate (PAPS) to the estrogenic molecule hydroxyl group. Sulfation with SULT1E1 increases the solubility of estrogens and, consequently, prevents the interaction between sulfated estrogens and the estrogen receptor. Estrogen sulfation is an irreversible process [29].

In an animal model, it has been shown that certain nutritional compounds induce SULT1A1 or SULT1E1, and at the same time, they are substrates for this enzyme. The main compounds are flavonoids—apigenin, epicatechin, resveratrol, chrysin, and quercetin [28,55].

## 7. Inflammatory and Oxidative Stress Biomarkers

Yousefi-Nodeh et al. (2022) showed that curcumin and vitamin E improve hot flashes in postmenopausal women. The presumed mechanism of action of curcumin and vitamin E is associated with the reduction in inflammation and oxidative stress [30].

In the early years of menopause, curcumin supplementation may improve indicators of oxidative stress such as total antioxidant capacity (TAC), malondialdehyde (MDA), and biomarker of inflammation high-sensitivity C-reactive protein (hs-CRP) [31]. The exact molecular mechanism has not been determined, but, for example, the protective effects of curcumin in age-related cellular senescence are accomplished by inhibiting oxidative stress through upregulation of SIRT1 and NRF2 and downregulation of the p53/p21 pathway [57].

Vitamin E supplementation may also improve antioxidant status in the early years of menopause by increasing TAC levels [31]. The antioxidant effect of vitamin E is described in detail in the article of Miyazawa et al. (2019), but the molecular mechanism of this process in vasomotor dysfunctions is unknown [58].

## 8. Glucose Availability

Information on the relationship between blood glucose levels and hot flashes is sparse and dates back more than a decade. Nevertheless, due to the significant share of glucose in the diet, they seem very important and worth presenting.

In his review article, Dormire (2009) presents the hypothesis that hot flashes are an indicator of transient glucose deficiencies in the central nervous system, secondary to reduced estrogen stimulation of glucose transporter 1 (GLUT 1). Low estrogen levels that occur during menopause result in reduced glucose availability in neuroendothelial cells in ovariectomized rats. As has been shown in studies in animal models, hot flashes can be triggered by stimuli that, for example, lower blood glucose levels or block the ability of brain cells to use glucose. According to the current literature data, research on the molecular pathways of glucose transport mediated by GLUT receptors and their involvement in generating hot flashes was not continued [59].

Estrogens, through their direct action on various nervous systems, bidirectionally modulate cognitive functions, which require an energy load on the brain and, at the same time, an adequate supply of metabolic substrates, such as glucose, lactates, and ketones, to function efficiently [60,61,62]. Reduced estrogen levels during menopause are probably associated with a deficit in glucose availability, which in turn leads to dysregulation of energy homeostasis and increases the risk of nervous disorders. As demonstrated in a mouse model of Alzheimer’s disease, ovariectomy resulted in reduced glucose uptake by the central nervous system and a shift in the bioenergetic profile in the hippocampus to one that favors reduced glucose utilization and increased lactate and ketone utilization [63].

## 9. Racial/Ethnic Conditions

It has also been suggested that racial/ethnic patterns may be related to genetic variation as an underlying mechanism causing vasomotor symptoms. There is a hypothesis that there are genetic variants that may be associated with the occurrence of VMS. However, the small number of candidate genes and GWAS studies performed, the small sample size of most studies, and the ambiguous results for the same genetic variants should be taken into account. All this together justifies the need for further research on this topic. As has been shown in studies in US women, the incidence, duration, and severity of VMS are highest in African American women, lowest among Asian women, and intermediate among white women of Hispanic and non-Hispanic descent. The best-studied and documented variant of the CYP1B1 gene is the rs1056836 variant, which is associated with a 40% lower likelihood of moderate to severe hot flashes among African American women [15,64]. The protein product of the CYP1B1 gene is known to be involved in the metabolism of endogenous compounds, regulation of metabolism, accumulation, and distribution of adipose tissue. It also takes part in the metabolism of arachidonic acid, which may lead to obesity and insulin resistance. CYP1B1 expression increases under adipogenesis stimulation [54]. Food components that influence CYP1B1 expression are discussed in the section on cellular signaling in the aryl hydrocarbon receptor pathway.

## 10. Conclusions

**Figure 1:**
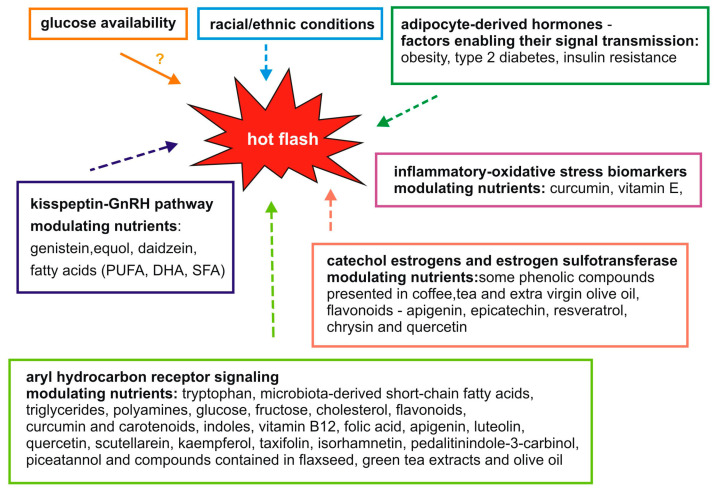
Proposed molecular mechanisms and food components modulating them that may potentially influence hot flashes.

Menopause is associated with the occurrence of many physiological and psychological symptoms that significantly affect women’s health, well-being, and quality of life. Hot flashes are one of the most troublesome symptoms for women during menopause. However, the mechanisms underlying hot flashes are not fully understood. Therefore, potential processes and signaling pathways are being sought at the molecular level, the modulation of which could lead to the alleviation of the symptoms of hot flashes. Literature data suggest that hot flashes may be associated not only with a decrease in estrogen levels or processes regulated by the hypothalamus but also with adipocyte-derived hormones, the kisspeptin-GnRH pathway, aryl hydrocarbon receptor signaling, glucose availability, inflammatory–oxidative stress and catechol estrogens and estrogen sulfotransferase. The importance of diet in the maintenance of human health is supported by abundant evidence [65]. In the present review, we have also focused on many food-derived nutrients and their roles as modulators of potential molecular mechanisms that may be related to menopausal hot flashes. A summary of these findings from published studies is shown in Figure 1. Detailed studies of these relationships may contribute to the development of new therapies for hot flashes in the future.

## Figures and Tables

**Table 1:** Nutritional compounds modulating cell signaling pathways that may potentially be involved in mechanisms causing hot flashes (in vitro and in vivo models).

Compound Name, Concentration Used	Model	Property	Chemical Structure, IUPAC Name	Reference
kisspeptin-GnRH (gonadotropin-releasing hormone) pathway-modulating nutrients				
(±)-equol 0–20 µmol/L	GT1-7 cells (mouse hypothalamic GnRH neuronal cell line)	increase in gonadotropin-releasing hormone secretion, and disruption of neurokinin B receptor expression	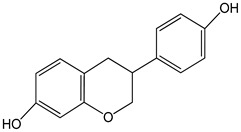 (3*S*)-isoflavan-4,7′-diol	[18]
genistein 0–80 µM for 48 h	GT1-7 cells (mouse hypothalamic GnRH neuronal cell line)	increase in GnRH secretion	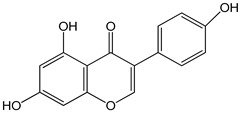 (5,7-dihydroxy-3-(4-hydroxyphenyl)chromen-4-one)	[19]
DHA (docosahexaenoic acid) 100 µM	mHypoA-59 cell line (primary hypothalamic culture isolated from 2-month old NPY-GFP mice)	upregulation of GnRH mRNA expression	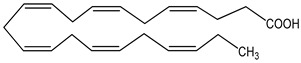 (docosahexaenoic acid; *cis*-4,7,10,13,16,19-docosahexaenoic acid)	[20]
PUFA (polyunsaturated fatty acid) 100 µM	mHypoA-59 cell line (primary hypothalamic culture isolated from 2-month old NPY-GFP mice)	upregulation of GnRH mRNA expression	linoleic acid as representative 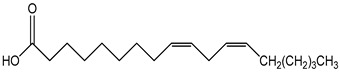 (cis, cis-9,12-octadecadienoic acid)	[20]
SFA (saturated fatty acid) 100 µM	mHypoA-59 cell line (primary hypothalamic culture isolated from 2-month old NPY-GFP mice)	upregulation of GnRH mRNA expression	palmitic acid as epresentative 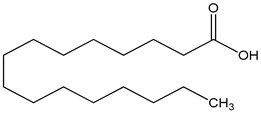 (hexadecanoic acid)	[20]
undernutrition feed-restricted to lose 20% of pre-study body weight over 13 weeks	Suffolk ewe lambs (fifteen lambs from single, twin, or triplet pregnancies; age 4–5 months)	decreased kisspeptin mRNA expression within kisspeptin-neurokinin B-dynorphin (KNDy) neurons		[21]
aryl hydrocarbon receptor (AHR) signaling-modulating nutrients				
β-carotene 1 µM	HepG2 (hepatoma) cell line	aryl hydrocarbon receptor (AHR) activation	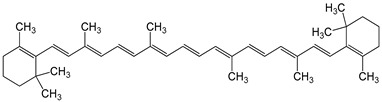	[22]
curcumin 1 µM	astrocyte culture derived from cerebral cortices collected from 2-day-old Sprague Dawley rats HepG2 (hepatoma) cell line	aryl hydrocarbon receptor (AHR) activation	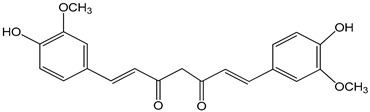 ((E,E)-1,7-bis(4-hydroxy-3-methoxyphenyl)-1,6-heptadiene-3,5-dione)	[22,23]
folic acid (pteroyl-L-glutamic acid) folic acid-deficient diet	HepG2 (hepatoma) cell line and C57BL/6 (B6) mice (B6 mice harboring the nonresponsive Ahr^d^ allele (AhR null))	suppression of aryl hydrocarbon receptor (AHR) transcriptional activity	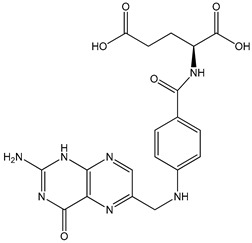 *N*-(4-{[(2-amino-4-oxo-3,4-dihydropteridin-6-yl)methyl]amino}benzoyl)-L-glutamic acid	[24]
high-fat diet (HFD) components: cholesterol (5-cholesten-3β-ol) (10 µg/mL), fructose (25 mM), palmitic acid (5 µM)	human aortic endothelial cells (HAEC) and human U937-derived macrophages (Umac)	aryl hydrocarbon receptor (AHR) activation	cholesterol 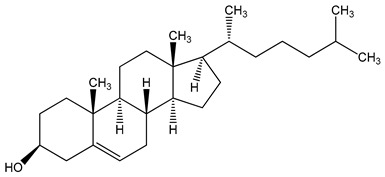 cholesterol (5-cholesten-3β-ol) 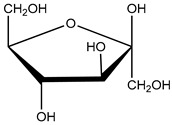 fructose	[25]
indole as tryptophan metabolite		aryl hydrocarbon receptor (AHR) activation	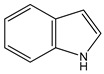 (1*H*-benzo[*b*]pyrrole)	[26]
palmitate 100 µM	mHypoA-GnRH/GFP cell line (generated from the hypothalamus of a 2-month old transgenic GnRH-GFP female mouse)	modulation of spexin, and its receptors Galr2 and Galr3, in GnRH neurons	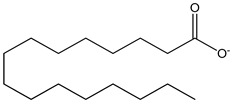	[27]
tryptophan metabolites AIN93M standard diet for rodents supplemented with 0.5% tryptophan	female C57BL/6 WT and KO mice	aryl hydrocarbon receptor (AHR) activation	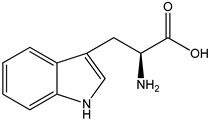 ((*S*)-2-amino-3-(3-indolyl)propionic acid)	[26,28]
vitamin B12 B12-deficient diet	HepG2 (hepatoma) cells and C57BL/6 (B6) mice (B6 mice harboring the nonresponsive Ahr^d^ allele (AhR null))	suppression of aryl hydrocarbon receptor (AHR) transcriptional activity	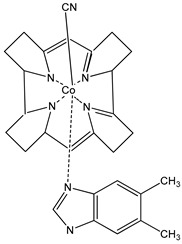 α-(5,6-dimethylbenzimidazolyl)cobamidcyanide	[24]
catechol estrogens and estrogen sulfotransferase-modulating nutrients				
apigenin 0–50 µM	*Sf9* cell line derived from pupa ovarian tissue of a fall armyworm; MCF-7 breast cancer cells	sulfotransferase family 1A member 1 (SULT1A1) induction	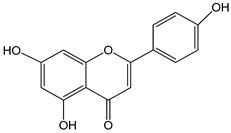 (5,7-dihydroxy-2-(4-hydroxyphenyl)-4-benzopyrone)	[29]
chrysin 0–10 µM	*Sf9* cell line derived from pupa ovarian tissue of a fall armyworm MCF-7 breast cancer cells	sulfotransferase family 1E member 1 (SULT1E1) induction	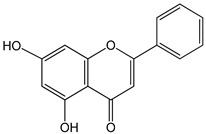 (5,7-dihydroxyflavone)	[29]
epicatechin 0–3000 µM	*Sf9* cell line derived from pupa ovarian tissue of a fall armyworm MCF-7 breast cancer cells	sulfotransferase family 1A member 1 (SULT1A1) activation	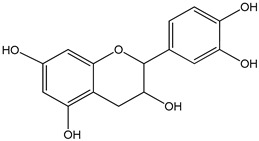 ((−)-*cis*-3,3′,4′,5,7-pentahydroxyflavane)	[29]
quercetin 0–10 µM	*Sf9* cell line derived from pupa ovarian tissue of a fall armyworm MCF-7 breast cancer cells	sulfotransferase family 1E member 1 (SULT1E1) activation	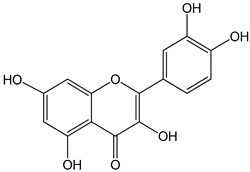 (2-(3,4-dihydroxyphenyl)-3,5,7-trihydroxychromen-4-one)	[29]
resveratrol 0–100 µM	*Sf9* cell line derived from pupa ovarian tissue of a fall armyworm MCF-7 breast cancer cells	sulfotransferase family 1A member 1 (SULT1A1) activation	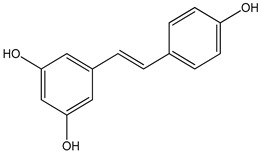 (3,4′,5-trihydroxy-*trans*-stilbene)	[29]
inflammatory–oxidative stress biomarker-modulating nutrients				
vitamin E 200 international units of alpha-tocopherol/d/8 weeks	postmenopausal women	reduction in hot flash episodes during the week improve the antioxidant status by increasing the total antioxidant capacity (TAC) levels	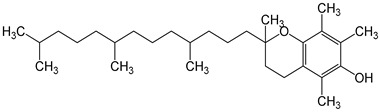	[30,31]

## Data Availability

Data sharing is not applicable.
